# Why does drug resistance readily evolve but vaccine resistance does not?

**DOI:** 10.1098/rspb.2016.2562

**Published:** 2017-03-29

**Authors:** David A. Kennedy, Andrew F. Read

**Affiliations:** Center for Infectious Disease Dynamics, Departments of Biology and Entomology, The Pennsylvania State University, University Park, PA, USA

**Keywords:** antimicrobial resistance, vaccine escape, pathogen evolution, evolutionary rescue

## Abstract

Why is drug resistance common and vaccine resistance rare? Drugs and vaccines both impose substantial pressure on pathogen populations to evolve resistance and indeed, drug resistance typically emerges soon after the introduction of a drug. But vaccine resistance has only rarely emerged. Using well-established principles of population genetics and evolutionary ecology, we argue that two key differences between vaccines and drugs explain why vaccines have so far proved more robust against evolution than drugs. First, vaccines tend to work prophylactically while drugs tend to work therapeutically. Second, vaccines tend to induce immune responses against multiple targets on a pathogen while drugs tend to target very few. Consequently, pathogen populations generate less variation for vaccine resistance than they do for drug resistance, and selection has fewer opportunities to act on that variation. When vaccine resistance has evolved, these generalities have been violated. With careful forethought, it may be possible to identify vaccines at risk of failure even before they are introduced.

## 1. Introduction

**Figure 1.**
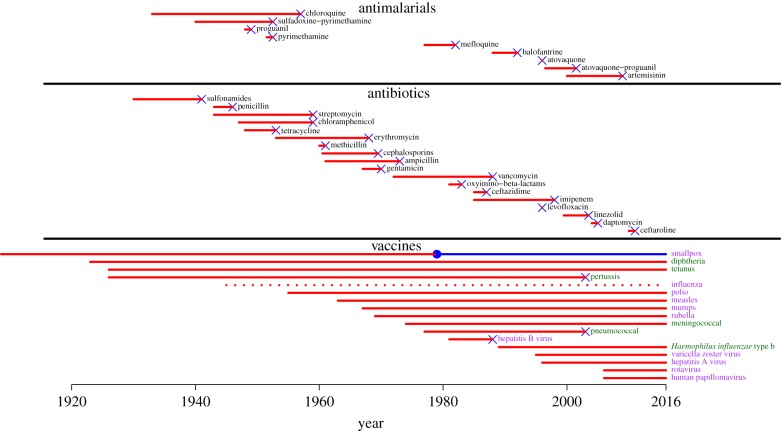
Time to first detection of human pathogens resistant to vaccines [1–6] and antimicrobial drugs [7]. Similar patterns exist for antiviral drugs, although antiviral resistance evolution can often be slowed by the use of combination antiviral therapy [8,9]. Viral vaccines are labelled in purple, bacterial vaccines are labelled in green. Blue ‘x's denote the first observations of resistance, with lines starting at product introduction (except for smallpox vaccination which began much earlier). Note that in all cases, substantial public health gains continued to accrue beyond the initial appearance of resistance. Only vaccines in the current immunization schedule recommended by the Centers for Disease Control and Prevention [6] are shown, with the addition of the smallpox vaccine. Global eradication of smallpox (marked as a filled, blue circle), ended the opportunity for resistance to emerge (blue line). The seasonal influenza vaccine is routinely undermined by antigenic evolution, evolution that occurs even in the absence of vaccination (dotted line). We took the earliest appearance of a vaccine-resistant pertussis variant to be the first record of a pertactin-negative strain [5]. This date [10] and several others (e.g. [11]) could be debated, but the general pattern is robust: resistance to drugs occurs more readily than resistance to vaccines.

Pathogen evolution impacts the efficacy of vaccines and antimicrobial drugs (e.g. antibiotics, antivirals, antimalarials) very differently (figure 1). After a new drug is introduced, drug resistance can rapidly evolve, leading to treatment failures [12]. For instance, most *Staphylococcus aureus* isolates in British hospitals were resistant to penicillin just 6 years after the introduction of the drug [13]. Similar evolutionary trajectories have been observed for the vast majority of drugs [14] and today many drugs are clinically useless against particular pathogens [15]. The problem has become so acute that drug resistance is viewed as one of the great challenges of our age, ranking alongside climate change and surpassing terrorism [16]. By striking contrast, vaccines generally provide sustained disease control. Most human vaccines have continued to provide protection since their introduction decades or even centuries ago (figure 1). For example, smallpox was eradicated because no virus strains capable of transmitting between vaccinated individuals ever emerged [17]. Indeed, the evolution of vaccine resistance is so rare that vaccines are now considered a leading solution to the drug resistance problem [11,18].

Yet drugs and vaccines both profoundly suppress pathogen fitness and so both should generate tremendous evolutionary pressure for resistance (defined here as a phenotype conferring increased pathogen replication or survival in treated hosts). Why then does pathogen evolution regularly undermine drug efficacy but rarely undermine vaccine efficacy (figure 1)? Here we propose that well-known principles of resistance management explain why vaccine resistance rarely evolves.

Note that we restrict our discussion to evolutionary changes that result either from mutation or from amplification of extremely rare variants (those maintained by mutation-selection balance). This focus excludes cases of ‘common-variant serotype replacement’ in which strains of a pathogen that were previously observed but intentionally not targeted by vaccines rise in frequency after the onset of vaccination. Although serotype replacement is a form of evolution, and an important consideration in a vaccinated host population, this process is perhaps better explained by purely ecological factors and thus warrants separate exploration [19]. To draw an analogy with drugs, serotype replacement is similar to an opportunistic infection like *Clostridium difficile* appearing after drugs were used to treat a different pathogen. That is undoubtedly an important phenomenon, but it is distinct from the evolution of resistance given that the intervention is still effective against its intended target.

A growing body of evidence suggests that the targets of several human vaccines are evolving (e.g. [10,20–23]), although the public health consequences of these evolutionary trajectories have often been unclear (e.g. [10,22,24–26]). Veterinary vaccines offer more examples, including the evolution of novel serotypes [27], antigenic loss [28], antigenic drift [29,30] and life-history modifications [31,32]. Nevertheless, vaccine resistance is relatively rare, and when it does emerge, it tends to take longer than antimicrobial resistance (figure 1).

It is well known that evolutionary trajectories are influenced by system-specific details. But there is a generality here: pathogen evolution almost always undermines drugs but rarely undermines vaccines (figure 1). This suggests that important features might be shared within each of these classes of disease intervention. For example, it is common to associate drugs with bacterial diseases and vaccines with viral diseases, and so one might wonder whether bacteria are simply more able to evolve resistance than viruses. But that cannot be a general explanation: viruses rapidly evolve resistance to antiviral drugs. For example, resistance to influenza [33,34] and herpesvirus drugs [35,36] emerged within a few years of FDA approval, and resistance to antivirals rapidly arises within human immunodeficiency virus (HIV) and hepatitis C virus (HCV)-infected patients unless they strictly adhere to certain treatment protocols [8,9] (a point to which we return below). Moreover, vaccine resistance has yet to emerge in several bacteria species (figure 1) even though drug resistance readily does. So it cannot be that drugs are more vulnerable to pathogen evolution because of some difference between bacteria and viruses. The explanation must lie elsewhere.

Previous efforts to understand the absence of vaccine resistance have mostly focused on measles. Frank & Bush [37] hypothesized that the inability of measles virus to escape vaccination might result from a trade-off between rapid pathogen transmission and antigenic flexibility. However, one might wonder why selection did not push this trade-off to favour antigenic flexibility once mass vaccination began to drive local extinction. Kalland *et al*. [38] suggested that measles virus might have an unusually low mutation rate for an RNA virus, but Schrag *et al*. [39] showed that measles virus mutates at rates similar to that of other RNA viruses. Fulton *et al*. [40] showed that measles virus antigens may be strongly constrained by natural selection, but in the same paper they also showed that evolutionary constraint is weaker for influenza virus antigens, suggesting that while antigenic constraint might be a property of measles virus, it is not an inherent property of vaccine targets.

We are aware of only one attempt to find a general explanation for why vaccine resistance is rare. McLean [41,42] observed that vaccines against childhood diseases like measles, polio and smallpox mimic natural immunity which pathogen evolution failed to evade despite intense selection for at least thousands of years (indeed, that is why they are called childhood diseases: they are restricted to non-immune individuals). She argues that natural and vaccine-induced immunity against these diseases are robust to pathogen evolution because they are ‘broadly cross reactive’. This raises the question of precisely what is meant by broadly cross reactive. Antimicrobial drugs are also broadly cross reactive in the sense that they too kill a wide variety of strains, yet drug resistance readily evolves.

Here we argue that vaccines are less vulnerable to pathogen evolution than are antimicrobial drugs because of differences in the way drugs and vaccines work. We contend that two key features of vaccines have large, synergistic effects on the rate at which resistance arises and then spreads (table 1, formalized in electronic supplementary material, appendix). Our hypothesis leans heavily on empirical and theoretical work designed to slow the evolution of drug resistance [43]. Elements of what we propose have been suggested before (e.g. [11,42,44–46]), but so far as we are aware, our argument has never appeared in its entirety.

**Table 1.** A summary of our argument. For the most part, vaccines act early and induce immunity which has multiple targets. These features reduce the likelihood of resistance originating in the first place and reduce the rate of spread of resistance if it does arise.

feature	origin	spread
early action (prophylaxis)	prophylaxis limits the accumulation of genetic diversity before intervention	pre-transmission clearance reduces opportunity for selection on partial resistance during spread
multiplicity of targets	combination-like effect reduces chance that resistance will appear	mosaic-like effect reduces the transmission advantage of resistance

## 2. Key factors

### (a) Timing of action

**Figure 2.**
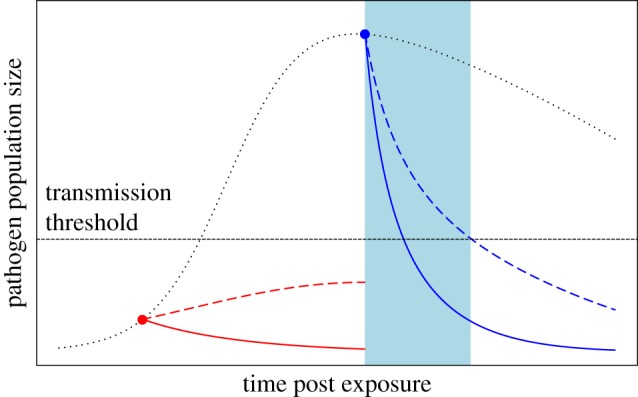
Schematic showing the effect of treatment timing on the evolution of resistance in a single infection. The dotted black line shows the pathogen population size over the course of an infection in an untreated host. The dashed black horizontal line shows the pathogen population size necessary for transmission. Dots mark the start of treatment, with red depicting early treatment (nearly prophylactic) and blue depicting later treatment (therapeutic). In comparison with later treatment, pathogen population size is small at the start of early treatment, reducing the likelihood that resistance will be generated *de novo*. In addition, when treatment is started sufficiently early, the sensitive pathogen population size (solid red curve) may never approach the threshold necessary for transmission and that might remain true even with small or moderate increases in resistance (dashed red curve). When treatment is started later, however, the sensitive pathogen (solid blue curve) may already be capable of transmission, so that small or moderate increases in resistance (dashed blue curve) would likely extend the window of time that the pathogen is transmissible. This creates a window of opportunity for partial resistance to be selectively favoured during spread to other hosts (shaded blue interval) that is not present when treatment begins early.

For most infectious diseases, hours to days elapse between exposure to a pathogen and symptomatic infection in a host. Typically, relatively few pathogen virions or cells establish an infection but then, as replication proceeds, populations balloon to the vast numbers associated with illness and infectiousness (e.g. [47–49]). Pathogen replication during this incubation period creates opportunities for mutations to arise, while pathogen transmission after this incubation period creates opportunities for these mutations to spread to new hosts. Therapies that act early can, therefore, be more robust to pathogen evolution because they limit replication and reduce the opportunities for spread to new hosts (table 1, figure 2).

The evolutionary benefit of treating infections early was noted over a century ago [50], but to reduce costs and side effects, drugs are typically administered therapeutically, meaning only after symptoms of disease arise. At the start of therapeutic treatment, the pathogen population within a host can be enormous, having already accumulated genetic diversity and become transmissible. Indeed, empirical studies have shown that the larger a microbe population is at the time of treatment, the more likely is the evolution of drug resistance [51]. This risk of therapeutic treatment is exacerbated during transmission between treated hosts, because differences in clearance rates between pathogen lineages could allow partially resistant lineages to transmit longer after treatment than less resistant ones (figure 2).

In contrast with drugs, vaccines are almost always used prophylactically. Prophylactic treatment, or the ongoing use of an intervention prior to known exposure, is the extreme limit of early treatment. The protective immune responses that vaccines elicit tend to keep pathogen populations from ever achieving large sizes, reducing the accumulation of diversity and opportunities for onward transmission. For example, tuberculosis vaccination suppresses peak bacterial population size 2–5 orders of magnitude in a rodent model [52]. Similarly, measles vaccination reduces virus titres by at least 3 orders of magnitude in a non-human primate model [53]. Pertussis vaccination reduces transmission from vaccinated hosts that become infected by 85% [54]. By keeping pathogen populations small and reducing onward transmission, potentially several orders of magnitude less diversity is interrogated by vaccine-induced immune selection than by drug-induced selection (figure 2). The prophylactic nature of vaccines thus reduces the opportunities for resistance to emerge and spread (table 1).

Drugs are not always used therapeutically, however. There are several examples where the prophylactic use of drugs has successfully prevented drug resistance. Perhaps, the clearest is the use of monotherapy with the antiretroviral zidovudine to prevent HIV infection in healthcare workers. This therapy routinely leads to evolution of resistance when given to HIV-infected patients [8], but when used as a post-exposure prophylaxis, it reduced infection risk in healthcare workers fivefold [55]. Nevertheless, prophylactic use can sometimes facilitate the evolution of drug resistance [56]. This is because prophylaxis can favour the spread of resistance once it has emerged in a population [57]. When contemplating the evolutionary risks of prophylactic drug use, it is thus crucial to understand whether resistance is already present within a population (or within a coexisting population that can donate genetic material).

### (b) Multiplicity of therapeutic targets within and between hosts

A second point is that the evolution of resistance may be slowed by therapeutic redundancy, whereby a microbial population is controlled in multiple efficacious ways. A vast literature has demonstrated that when multiple drugs are available, the way that these drugs are administered can affect the speed with which resistance evolves [12,43,58]. Two promising strategies are combination therapy (the simultaneous use of different drugs in the same host) and treatment mosaics (the simultaneous use of different drugs in different hosts) [43,59–63]

The benefit of combination therapy is based on the premise that resistance can only be acquired by the simultaneous acquisition of resistance to each component drug. The probability of simultaneous acquisition becomes vanishingly small as the number of drugs increases [12]. Combination therapy has improved outcomes for HIV and tuberculosis patients, largely by preventing the within-host evolution of drug resistance when patients are fully compliant [64,65]. The benefit of treatment mosaics is based on the premise that mosaics create heterogeneity in selection. Resistance to a particular drug is beneficial only in the fraction of hosts treated with that drug, and so the fitness advantage of that resistance plays out in fewer hosts when some hosts are treated with alternate drugs (electronic supplementary material, appendix). In cases where resistance imposes a substantial fitness cost or where resistance to one drug is associated with increased sensitivity to another (collateral sensitivity), selection in a mosaic might even favour sensitive pathogens over those resistant to a subset of drugs. Combination therapy thus reduces the chance that a resistant pathogen will emerge, and treatment mosaics slow or prevent the spread of resistance once it has emerged [43].

The logic behind the resistance management benefits of drug combinations and mosaics provides a second reason why vaccine resistance is rarer than drug resistance. A drug often interferes with a specific step in a specific metabolic pathway. A vaccine, however, often exposes the host immune system to multiple pathogen proteins (antigens), and multiple potential binding sites (epitopes) on each antigen [66]. Epitopes are recognized and bound by components of the immune system analogously to how biochemical molecules would interact with a drug or its downstream products. This means that immunity is in effect acting like combination therapy, but with substantially more component effectors (and hence targets) than any drug cocktail [66–68]. For example, approximately 100 unique tetanus-toxoid-specific antibodies can be observed in healthy humans after receiving a tetanus-toxoid booster vaccine, with these antibodies being unique between subjects [69]. The different antibody repertoires observed between subjects is due to the numerous mechanisms that generate antibody diversity during immune development [66,68]. Indeed, minimal overlap of immune repertoires is quite common [70] suggesting that vaccination also creates mosaic-like patterns in host populations. The high multiplicity of therapeutic vaccine targets thus reduces the chance that resistance will originate and the ability of resistance to spread should it originate (table 1).

The benefit of combination-like therapy in immune responses has been directly observed. For instance, influenza viruses readily evolve resistance to monoclonal antibodies *in vitro*, but the evolution of resistance is drastically reduced when different monoclonal antibodies are used simultaneously [71]. Similarly, simian immunodeficiency virus (SIV) quickly escapes rhesus macaque immunity when T-cell populations are dominated by a single T-cell receptor binding motif, but not when repertoires are more diverse [72]. Indeed, escape from monoclonal antibodies or monoclonal-antibody-like molecules is common *in vitro*, including for pathogens where vaccination has proved to be evolution resistant such as measles virus [39] and poliovirus [73]. We note that the benefits of multiple target sites would only be magnified if the antigens of such pathogens were functionally constrained by evolution, as they can apparently be [40].

## 3. Evolutionary declines in vaccine efficacy

It is our contention that vaccine resistance has evolved less often than drug resistance because (i) vaccines act early and (ii) vaccine-induced immunity generates potent multi-target attacks (table 1). Together, these features drastically increase the time until resistance emerges (electronic supplementary material, appendix). Nevertheless, pathogen evolution has reduced the efficacy of some vaccines. In this section, we argue that the benefits conferred by one or both of these features were missing for the three human vaccines where resistance is known to have emerged. Similar patterns are found for the documented cases of resistance evolution against animal vaccines.

The best documented example of vaccine resistance evolution occurred in Marek's disease, a commercially important disease of chickens caused by Marek's disease virus (*Gallid herpesvirus II*). There, two generations of vaccines were undermined by viral evolution [30]. Those vaccines prevented disease, but even before the pathogen evolved, they did not prevent viral infection, replication, or transmission. Instead, Marek's disease virus reached large population sizes even within vaccinated hosts and was able to transmit to new hosts. As a result, the virus was likely able to generate genetic diversity within vaccinated hosts, and vaccine-induced selection was able to act during transmission between hosts [32]. The benefits of prophylaxis were thus missing. Similar mechanisms likely explain two vaccine breaks by feline calicivirus [74], and a decline in efficacy of a vaccine against the malaria parasite *Plasmodium chabaudi* in a serial passage experiment in mice [75]. In each of these cases, vaccination provided substantial protection against disease, but even before evolution took place, vaccination was unable to completely prevent pathogen colonization, replication and transmission, creating opportunities for pathogen evolution.

Another well-documented example of vaccine resistance occurred in the bacteria *Yersinia ruckeri*, which causes enteric redmouth disease in farmed salmonids. Outbreaks of disease in vaccinated populations were traced to a new biotype characterized by its lack of flagella and phospholipase secretion activity [76]. In four separate lineages, the development of this new biotype was attributed to single mutational events that differed between the lineages but that acted in the same flagellar secretion pathway [28]. Thus, the high-multiplicity-of-target-sites benefit of vaccination was absent here: a single mutational event was capable of generating resistance to many mechanisms of action simultaneously. This is analogous to a mutation that confers cross resistance eliminating the benefit of combination drug therapy.

The human pathogen *Streptococcus pneumoniae* has shown evidence of evolution after the introduction of the pneumococcal conjugate vaccine. This vaccine elicits immunity against the capsular polysaccharide of the bacteria, reducing the probability of colonization with vaccine serotypes [77]. Even before the vaccine was deployed, there was speculation that vaccine-induced immunity would drive non-vaccine-targeted serotype replacement [78] and indeed, serotype replacement was duly observed [79]. But that was only part of the story. One of the serotypes targeted by the vaccine was able to escape vaccine immunity through recombination with a non-vaccine-targeted serotype [1]. Thus, the pathogen was able to evolve vaccine resistance because a single or small number of recombination events allowed the pathogen to simultaneously sidestep effectors against multiple targets on the capsular polysaccharide.

The rapid appearance of vaccine resistance in the human pathogen hepatitis B virus (HBV) can be interpreted in a similar way. Recombinant vaccines have successfully reduced disease incidence, but even shortly after the introduction of vaccination, mutant strains of HBV were observed in vaccinated hosts [2]. The first reported case of vaccine resistance occurred in Italy shortly after the vaccine was introduced [80]. In this system, immune protection is conferred almost entirely by immune recognition of a single conformational antigen that consists of only nine amino acids [26]. Several single nucleotide mutations have been shown to confer immune escape [81]. Vaccination in this system thus lacks multiple targets.

The human bacterial pathogen *Bordetella pertussis* has also shown evidence of vaccine-driven evolution. Some authors contend that this may partially explain the resurgence of pertussis in vaccinated countries [10,22,24]. Initially, this evolution appeared consistent with strain replacement, where strains of pertussis dissimilar to vaccine strains increased in frequency [10,82]. However, the evolutionary trajectory appeared to change after acellular vaccines replaced the original whole cell vaccines. Acellular vaccines protect against disease, but may not prevent infection and transmission [83]. They also contain a subset of the antigens present in the whole cell vaccine. After acellular vaccines were introduced, strains of pertussis that did not produce pertactin [84], one of the few antigens in typical acellular vaccines, began to increase in frequency in several countries concurrently [85]. Consistent with the expectation of vaccine-driven evolution, pertactin-negative strains were more common in vaccinated than unvaccinated patients [86]. Moreover, pertactin-negative strains have a competitive advantage over pertactin-positive strains in vaccinated mice [87]. It is also possible that *Bordetella* stains producing more immunosuppressive pertussis toxin have evolved in response to acellular vaccine-induced immunity [22]. The lack of complete pathogen clearance during the incubation period may partially explain both evolutionary trajectories; the situation was probably exacerbated because the acellular vaccines elicit immunity against so few targets.

## 4. Discussion

We have argued that drug resistance has tended to evolve faster than vaccine resistance (figure 1) because, for the most part, drugs are used therapeutically whereas vaccines are used prophylactically, and drugs attack far fewer target sites than do vaccines. This means that drug resistance is more likely to arise in the first place and then spread more rapidly once it has arisen (table 1). By contrast, vaccines prevent infection and transmission and induce immunity against many pathogen target sites, making it hard for vaccine resistance to evolve. In the electronic supplementary material, appendix, we show that together, both features have a substantially stronger impact than either alone. The few examples of vaccine-associated evolution detected in human and animal pathogens have occurred against vaccines that lack the benefits of one or both of these features.

### (a) Non-key features

Vaccines and drugs differ in many ways. But unlike the timing of action and the multiplicity of target sites which have large effects across a wide range of infectious diseases, we believe other features of drugs and vaccines are likely to have at best only moderate or system-specific effects on the rate of resistance evolution.

One set of differences between vaccines and drugs stem from the fact that vaccine effects are mediated through host immune responses while drugs effects are mediated through chemical pathways. This has at least six consequences. First, vaccines do not interact directly with pathogens, but instead act indirectly. Whether this reduces the ability of the pathogen population to evolve is unclear, but resistance is seen against drugs such as solfonamides that also act indirectly (figure 1). Second, vaccines induce systemic host responses that may minimize spatial refugia and spatial heterogeneity within hosts. In some cases, refugia increase opportunities for resistance to emerge by providing a continuous source of genetic variants to probe the therapeutic environment. Heterogeneity can also provide a gradient of therapeutic strength over which selection can act. Nevertheless, drug resistance readily evolves *in vitro*, a setting that minimizes spatial variation in drug dose. Third, immune responses are outside the control of individual patients, reducing opportunities for non-compliance that may create temporal heterogeneities and temporal refugia within hosts. However, resistance can evolve against drugs even when patients are fully compliant (e.g. [88]). Fourth, vaccines are only active while pathogens are inside hosts, but drugs can remain active in environmental reservoirs [89], suggesting that the strength of selection for resistance may differ for drug and vaccine resistance. However, drug resistance readily evolves even in pathogens that lack environmental life stages such as HIV [8]. Fifth, the immune system tends to be highly pathogen-specific and so vaccines are in effect, more-narrow spectrum than most antimicrobial drugs. Antimalarial drugs, however, have an extremely narrow microbial target range in practice, yet resistance is widespread [7]. Sixth, host immune systems have been shaped by coevolution between pathogens and hosts. However, microorganisms, have also been coevolving with drug effectors long before the medical use of drugs or vaccines [90], and indeed longer than they have dealt with vertebrate immunity. Moreover, it is not clear that the age of arms races predicts which party, if either, will win them.

Another difference between drugs and vaccines is that vaccination indirectly reduces total pathogen population size through herd immunity. We argued above that pathogen population size within a host is a key factor in the rate of resistance evolution, because vaccines target pathogen populations when they are orders of magnitude below maximal. Herd immunity can also reduce pathogen population sizes by orders of magnitude, but this decrease is unlikely to have a similar influence on the rate at which resistance evolves. This is because during the rollout of a vaccination strategy intended for the elimination of an endemic pathogen, vaccinated hosts will frequently contact the pathogen creating opportunities for selection on resistance. The effect of herd immunity on the emergence of vaccine resistance will therefore have little effect until later into a vaccination campaign when infection prevalence is substantially reduced. This rollout period often extends beyond 5–10 years after which drug resistance is commonly observed. For example, pertussis cases in Massachusetts decreased by 2 orders of magnitude only after 20 years of routine vaccination [91].

Biases in observing resistance to drugs and vaccines might also contribute to the differences in the rate at which resistance is first seen. For example, the processes of hypermutation and affinity maturation might generate short-term robustness to vaccine resistance [66]. If resistance mutants were being obscured by this process, however, serum neutralization ability post vaccination against circulating pathogen isolates should have decreased over time. Alternatively, there may be biases during the selection and development of targets for drugs and vaccines. For example, vaccines might have been developed only against pathogens with little potential for antigenic evolution. This pattern might occur if evolution differentially undermines candidate drugs and vaccines during clinical trials. It might also result from unknown selection bias in choosing targets for vaccination. If they exist, we suggest that any such patterns might be mechanistically explained by our main argument that the prophylactic and multi-target nature of vaccines inhibits the ability of pathogens to evolve resistance.

### (b) Serotype replacement

When a pathogen population consists of diverse serotypes, vaccination against a subset of serotypes can lead to an increase in the prevalence of others. This phenomenon of serotype replacement has occurred against several human vaccines [19]. Our framework is not intended to explain this type of evolution. We would not expect vaccines with early action and a high multiplicity of target sites to prevent serotype replacement. Understanding how serotype diversity changes following vaccination is a challenge [10,19,78,79], not least because theory on the maintenance of serotype diversity even in the absence of vaccination is still developing [92,93]. We note, however, that serotype replacement has not universally undermined vaccine efficacy, in part, because some vaccine targets do not have serotype diversity (e.g. measles virus) or because vaccination protects against all known serotypes (e.g. poliovirus).

### (c) Implications

Vaccines against some diseases have been notoriously difficult to develop [94]. Some of this difficulty is attributable to high antigenic variation as a result of immune selection (e.g. HIV, HCV, rhinoviruses, malaria). Indeed, immune selection driving rapid antigenic evolution is why the influenza vaccine must be regularly updated [95]. Recent vaccine development for several diseases has, therefore, focused on inducing the production of broadly neutralizing antibodies that target conserved rather than variable antigens [96]. Yet resistance to broadly neutralizing antibodies can rapidly evolve [97]. We argue that the multiplicity of antigens and epitopes targeted by broadly neutralizing antibodies is likely to be a key factor in determining whether resistance to such vaccines will evolve.

Concern about the long-term efficacy of chemotherapeutic drugs existed even before the mass distribution of the first antibiotics [50], but no such concerns were raised during the development of early vaccines. Of course, the first vaccine came well before Darwin, the germ theory of infection and indeed drugs, but now that humanity understands evolution, and has watched pathogen adaptation repeatedly undermine most antimicrobial drugs, it seems timely to ask whether past vaccine successes are a good indicator of future performance. Like McLean [41], we see no reason to be complacent. A growing body of evidence suggests that pathogens may be evolving in response to several vaccines currently in widespread use [1,2,10,20,22], and serotype replacement is clear in several cases [19]. We are concerned that pathogen adaptation may undermine existing and next generation vaccines that induce immune responses at rather few target sites and that fail to provide adequate control of replication and transmission. A substantial body of work is investigating ways to slow the evolution of drug resistance. Understanding why drug resistance is common and vaccine resistance is rare may help make next generation vaccines as resistant to evolutionary escape as their predecessors. The lessons learned might also give new insights into preventing the emergence of resistance to drugs, cancer immunotherapies and antimicrobial peptides.

## Supplementary Material

Appendix from Why does drug resistance readily evolve but vaccine resistance does not?

## References

[1] Brueggemann AB, Pai R, Crook DW, Beall B 2007 Vaccine escape recombinants emerge after pneumococcal vaccination in the United States. PLoS *Pathog.* 3, e168 (10.1371/journal.ppat.0030168) PMC2077903 18020702

[2] Carman WF, Karayiannis P, Waters J, Thomas HC, Zanetti AR, Manzillo G, Zuckerman AJ 1990 Vaccine-induced escape mutant of hepatitis B virus. Lancet 336, 325–329. (10.1016/0140-6736(90)91874-A) 1697396

[3] Hilleman MR 1998 Six decades of vaccine development—a personal history. Nat. Med. 4, 507–514. (10.1038/nm0598supp-507) 9585201

[4] Plotkin SL, Plotkin SA 2008 A short history of vaccination. In Vaccines: *fifth edition* (eds Plotkin SA, Orenstein WA, Offit PA), pp. 1–16. Philadelphia, PA: Elsevier Inc.

[5] Hegerle N, Paris AS, Brun D, Dore G, Njamkepo E, Guillot S, Guiso N 2012 Evolution of French *Bordetella pertussis* and *Bordetella parapertussis* isolates: increase of *Bordetellae* not expressing pertactin. Clin. Microbiol. Infect. 18, E340–E346. (10.1111/j.1469-0691.2012.03925.x) 22717007

[6] Centers for Disease Control and Prevention. 2015 Epidemiology and prevention *of vaccine-preventable diseases*, 13th edn (eds Hambrosky J, Kroger A, Wolfe C). Washington, DC: Public Health Foundation.

[7] McClure NS, Day T 2014 A theoretical examination of the relative importance of evolution management and drug development for managing resistance. Proc. R. Soc. B 281, 20141861 (10.1098/rspb.2014.1861) PMC4240990 25377456

[8] Larder BA, Darby G, Richman DD 1989 HIV with reduced sensitivity to zidovudine (AZT) isolated during prolonged therapy. Science 243, 1731–1734. (10.1126/science.2467383) 2467383

[9] Ke R, Loverdo C, Qi H, Sun R, Lloyd-Smith JO 2015 Rational design and adaptive management of combination therapies for hepatitis C virus infection. PLoS *Comput. Biol.* 11, e1004040 (10.1371/journal.pcbi.1004040) PMC4488346 26125950

[10] Mooi FR, van Oirschot H, Heuvelman K, van der Heide HGJ, Gaastra W, Willems RJL 1998 Polymorphism in the *Bordetella pertussis* virulence factors p. 69/pertactin and pertussis toxin in the Netherlands: temporal trends and evidence for vaccine-driven evolution. Infect. Immun. 66, 670–675. 9453625 10.1128/iai.66.2.670-675.1998 PMC107955

[11] Mishra RPN, Oviedo-Orta E, Prachi P, Rappuoli R, Bagnoli F 2012 Vaccines and antibiotic resistance. Curr. Opin. Microbiol. 15, 596–602. (10.1016/j.mib.2012.08.002) 22981392

[12] zur Wiesch PA, Kouyos R, Engelstädter J, Regoes RR, Bonhoeffer S 2011 Population biological principles of drug-resistance evolution in infectious diseases. Lancet Infect. Dis. 11, 236–247. (10.1016/S1473-3099(10)70264-4) 21371657

[13] Barber M, Whitehead JEM 1949 Bacteriophage types in penicillin-resistant staphylococcal infection. Br. Med. J. 2, 565 (10.1136/bmj.2.4627.565) 18148090 PMC2051145

[14] Davies J, Davies D 2010 Origins and evolution of antibiotic resistance. Microbiol. Mol. Biol. Rev. 74, 417–433. (10.1128/MMBR.00016-10) 20805405 PMC2937522

[15] MacGowan A, Albur M 2013 Frontline antibiotic therapy. Clin. Med. 13, 263–268. (10.7861/clinmedicine.13-3-263) PMC5922670 23760700

[16] Davies SC 2013 Annual Report of the Chief Medical Officer, Volume Two, 2011. London, UK: Department of Health.

[17] Fenner F 1983 The Florey lecture, 1983: biological control, as exemplified by smallpox eradication and myxomatosis. Proc. R. Soc. Lond. B 218, 259–285. (10.1098/rspb.1983.0039) 6136042

[18] Lipsitch M, Siber GR 2016 How can vaccines contribute to solving the antimicrobial resistance problem? mBio 7, e00428-16 (10.1128/mBio.00428-16) 27273824 PMC4959668

[19] Martcheva M, Bolker BM, Holt RD 2008 Vaccine-induced pathogen strain replacement: what are the mechanisms? J. R. Soc. Interface 5, 3–13. (10.1098/rsif.2007.0236) 17459810 PMC2405901

[20] Harrison TJ, Hopes EA, Oon CJ, Zanetti AR, Zuckerman AJ 1991 Independent emergence of a vaccine-induced escape mutant of hepatitis B virus. J. Hepatol. 13, S105–S107. (10.1016/0168-8278(91)90037-C) 1726588

[21] Schouls LM, van der Ende A, van de Pol I, Schot C, Spanjaard L, Vauterin P, Wilderbeek D, Witteveen S 2005 Increase in genetic diversity of *Haemophilus influenzae* serotype b (Hib) strains after introduction of Hib vaccination in the Netherlands. J. Clin. Microbiol. 43, 2741–2749. (10.1128/JCM.43.6.2741-2749.2005) 15956392 PMC1151946

[22] Octavia S, Sintchenko V, Gilbert GL, Lawrence A, Keil AD, Hogg G, Lan R 2012 Newly emerging clones of *Bordetella pertussis* carrying *prn2* and *ptxP3* alleles implicated in Australian pertussis epidemic in 2008–2010. J. Infect. *Dis.* 205, 1220–1224. (10.1093/infdis/jis178) 22416243

[23] Drexler JF et al. 2014 Robustness against serum neutralization of a poliovirus type 1 from a lethal epidemic of poliomyelitis in the Republic of Congo in 2010. Proc. Natl Acad. Sci. USA 111, 12 889–12 894. (10.1073/pnas.1323502111) PMC4156724 25136105

[24] Lam C et al. 2014 Rapid increase in pertactin-deficient *Bordetella pertussis* isolates, Australia. Emerg. Infect. Dis. 20, 626–633. (10.3201/eid2004.131478) 24655754 PMC3966384

[25] Clark TA 2014 Changing pertussis epidemiology: everything old is new again. J. Infect. Dis. 209, 978–981. (10.1093/infdis/jiu001) 24626532

[26] Romanò L, Paladini S, Galli C, Raimondo G, Pollicino T, Zanetti AR 2015 Hepatitis B vaccination: are escape mutant viruses a matter of concern? Hum. *Vaccin. Immunother.* 11, 53–57. (10.4161/hv.34306) PMC4514213 25483515

[27] Lu H, Tang Y, Dunn PA, Wallner-Pendleton EA, Lin L, Knoll EA 2015 Isolation and molecular characterization of newly emerging avian reovirus variants and novel strains in Pennsylvania, USA, 2011–2014. Sci. Rep. 5, 14727 (10.1038/srep14727) 26469681 PMC4606735

[28] Welch TJ, Verner-Jeffreys DW, Dalsgaard I, Wiklund T, Evenhuis JP, Cabrera JAG, Hinshaw JM, Drennan JD, LaPatra SE 2011 Independent emergence of *Yersinia ruckeri* biotype 2 in the United States and Europe. App. Environ. Microb. 77, 3493–3499. (10.1128/AEM.02997-10) PMC3126439 21441334

[29] Lee CW, Senne DA, Suarez DL 2004 Effect of vaccine use in the evolution of Mexican lineage H5N2 avian influenza virus. J. Virol. 78, 8372–8381. (10.1128/JVI.78.15.8372-8381.2004) 15254209 PMC446090

[30] Witter RL 1997 Increased virulence of Marek's disease virus field isolates. Avian *Dis.* 41, 149–163. (10.2307/1592455) 9087332

[31] Franzo G, Tucciarone CM, Cecchinato M, Drigo M 2016 Porcine circovirus type 2 (PCV2) evolution before and after the vaccination introduction: a large scale epidemiological study. Sci. Rep. 6, 39458 (10.1038/srep39458) 27991573 PMC5171922

[32] Read AF, Baigent SJ, Powers C, Kgosana LB, Blackwell L, Smith LP, Kennedy DA, Walkden-Brown SW, Nair VK 2015 Imperfect vaccination can enhance the transmission of highly virulent pathogens. PLoS Biol. 13, e1002198 (10.1371/journal.pbio.1002198) 26214839 PMC4516275

[33] Gubareva LV, Matrosovich MN, Brenner MK, Bethell RC, Webster RG 1998 Evidence for zanamivir resistance in an immunocompromised child infected with influenza B virus. J. Infect. Dis. 178, 1257–1262. (10.1086/314440) 9780244

[34] de Jong MD et al. 2005 Oseltamivir resistance during treatment of influenza A (H5N1) infection. New Engl. J. Med. 353, 2667–2672. (10.1056/NEJMoa054512) 16371632

[35] Linnemann CC, Biron KK, Hoppenjans WG, Solinger AM 1990 Emergence of acyclovir-resistant varicella zoster virus in an AIDS patient on prolonged acyclovir therapy. AIDS 4, 577–580. (10.1097/00002030-199006000-00014) 2167103

[36] Walling DM, Flaitz CM, Nichols CM 2003 Epstein–Barr virus replication in oral hairy leukoplakia: response, persistence, and resistance to treatment with valacyclovir. J. Infect. Dis. 188, 883–890. (10.1086/378072) 12964120

[37] Frank SA, Bush RM 2007 Barriers to antigenic escape by pathogens: trade-off between reproductive rate and antigenic mutability. BMC Evol. Biol. 7, 229 (10.1186/1471-2148-7-229) 18005440 PMC2217548

[38] Kalland KH, Håvarstein L, Endresen C, Haukenes G 1990 Stability of the nucleotide sequence of the phosphoprotein gene of measles virus during lytic infections. APMIS 98, 327–335. (10.1111/j.1699-0463.1990.tb01040.x) 2354052

[39] Schrag SJ, Rota PA, Bellini WJ 1999 Spontaneous mutation rate of measles virus: direct estimation based on mutations conferring monoclonal antibody resistance. J. Virol. 73, 51–54. 9847306 10.1128/jvi.73.1.51-54.1999 PMC103807

[40] Fulton BO, Sachs D, Beaty SM, Won ST, Lee B, Palese P, Heaton NS 2015 Mutational analysis of measles virus suggests constraints on antigenic variation of the glycoproteins. Cell Rep. 11, 1331–1338. (10.1016/j.celrep.2015.04.054) 26004185 PMC4464907

[41] McLean AR 1995 Vaccination, evolution and changes in the efficacy of vaccines: a theoretical framework. Proc. R. Soc. Lond. B 261, 389–393. (10.1098/rspb.1995.0164) 8587880

[42] McLean AR 1998 Vaccines and their impact on the control of disease. Br. *Med. Bull.* 54, 545–556. (10.1093/oxfordjournals.bmb.a011709) 10326283

[43] REX Consortium. 2013 Heterogeneity of selection and the evolution of resistance. Trends Ecol. Evol. 28, 110–118. (10.1016/j.tree.2012.09.001) 23040463

[44] Gandon S, Mackinnon MJ, Nee S, Read AF 2001 Imperfect vaccines and the evolution of pathogen virulence. Nature 414, 751–756. (10.1038/414751a) 11742400

[45] Frank SA 2002 Immunology and evolution of infectious disease. Princeton, NJ: Princeton University Press. 20821852

[46] Mackinnon MJ, Gandon S, Read AF 2008 Virulence evolution in response to vaccination: the case of malaria. Vaccine 26, C42–C52. (10.1016/j.vaccine.2008.04.012) 18773536 PMC2663389

[47] Holmes EC 2009 The evolutionary genetics of emerging viruses. Ann. Rev. *Ecol. Evol. Syst.* 40, 353–372. (10.1146/annurev.ecolsys.110308.120248)

[48] Gutiérrez S, Michalakis Y, Blanc S 2012 Virus population bottlenecks during within-host progression and host-to-host transmission. Curr. Opin. Virol. 2, 546–555. (10.1016/j.coviro.2012.08.001) 22921636

[49] Smith RC, Vega-Rodríguez J, Jacobs-Lorena M 2014 The *Plasmodium* bottleneck: malaria parasite losses in the mosquito vector. Mem. Inst. *Oswaldo Cruz* 109, 644–661. (10.1590/0074-0276130597) PMC4156458 25185005

[50] Ehrlich P 1913 Chemotherapeutics: scientific principles, methods and results. Lancet 2, 353–359.

[51] Ramsayer J, Kaltz O, Hochberg ME 2013 Evolutionary rescue in populations of *Pseudomonas fluorescens* across an antibiotic gradient. Evol. Appl. 6, 608–616. (10.1111/eva.12046) 23789028 PMC3684742

[52] Smith DW, Wiegeshaus E, Navalkar R, Grover AA 1966 Host–parasite relationships in experimental airborne tuberculosis I. Preliminary studies in BCG-vaccinated and nonvaccinated animals. J. Bacteriol. 91, 718–724. 4956758 10.1128/jb.91.2.718-724.1966 PMC314919

[53] Zhu Y, Heath J, Collins J, Greene T, Antipa L, Rota P, Bellini W, McChesney M 1997 Experimental measles. II. Infection and immunity in the rhesus macaque. Virology 233, 85–92. (10.1006/viro.1997.8575) 9229928

[54] Préziosi MP, Halloran ME 2003 Effects of pertussis vaccination on transmission: vaccine efficacy for infectiousness. Vaccine 21, 1853–1861. (10.1016/S0264-410X(03)00007-0) 12706669

[55] Cardo DM et al. 1997 A case–control study of HIV seroconversion in health care workers after percutaneous exposure. New Engl. J. Med. 337, 1485–1490. (10.1056/NEJM199711203372101) 9366579

[56] Cabello FC 2006 Heavy use of prophylactic antibiotics in aquaculture: a growing problem for human and animal health and for the environment. Environ. *Microbiol.* 8, 1137–1144. (10.1111/j.1462-2920.2006.01054.x) 16817922

[57] Kunkel A, Colijn C, Lipsitch M, Cohen T 2015 How could preventive therapy affect the prevalence of drug resistance? Causes and consequences. Phil. *Trans. R. Soc. B* 370, 20140306 (10.1098/rstb.2014.0306) PMC4424438 25918446

[58] Pena-Miller R, Laehnemann D, Jansen G, Fuentes-Hernandez A, Rosenstiel P, Schulenburg H, Beardmore R 2013 When the most potent combination of antibiotics selects for the greatest bacterial load: the smile–frown transition. PLoS Biol. 11, e1001540 (10.1371/journal.pbio.1001540) 23630452 PMC3635860

[59] Fish DN, Piscitelli SC, Danziger LH 1995 Development of resistance during antimicrobial therapy: a review of antibiotic classes and patient characteristics in 173 studies. Pharmacotherapy 15, 279–291. 7667163

[60] Bonhoeffer S, Lipsitch M, Levin BR 1997 Evaluating treatment protocols to prevent antibiotic resistance. Proc. Natl Acad. Sci. USA 94, 12 106–12 111. (10.1073/pnas.94.22.12106) PMC23718 9342370

[61] Bergstrom CT, Lo M, Lipsitch M 2004 Ecological theory suggests that antimicrobial cycling will not reduce antimicrobial resistance in hospitals. Proc. Natl Acad. Sci. USA 101, 13 285–13 290. (10.1073/pnas.0402298101) PMC516561 15308772

[62] Boni MF, Smith DL, Laxminarayan R 2008 Benefits of using multiple first-line therapies against malaria. Proc. Natl Acad. Sci. USA 105, 14 216–14 221. (10.1073/pnas.0804628105) PMC2544604 18780786

[63] Nguyen TD, Olliaro P, Dondorp AM, Baird JK, Lam HM, Farrar J, Thwaites GE, White NJ, Boni MF 2015 Optimum population-level use of artemisinin combination therapies: a modelling study. Lancet Glob. Health 3, e758–e766. (10.1016/S2214-109X(15)00162-X) 26545449 PMC4641185

[64] Chesney MA, Morin M, Sherr L 2000 Adherence to HIV combination therapy. Soc. Sci. Med. 50, 1599–1605. (10.1016/S0277-9536(99)00468-2) 10795966

[65] Gillespie SH 2002 Evolution of drug resistance in *Mycobacterium tuberculosis*: clinical and molecular perspective. Antimicrob. Agents Chemother. 46, 267–274. (10.1128/AAC.46.2.267-274.2002) 11796329 PMC127054

[66] Janeway CA, Travers P, Walport M, Capra JD 2005 Immunobiology: the immune system in health and disease. New York, NY: Garland Science.

[67] Davis MM, Bjorkman PJ 1988 T-cell antigen receptor genes and T-cell recognition. Nature 334, 395–402. (10.1038/334395a0) 3043226

[68] Alberts B, Johnson A, Lewis J, Raff M, Roberts K, Walter P 2002 Molecular biology of the cell. New York, NY: Garland Science.

[69] Poulsen TR, Meijer PJ, Jensen A, Nielsen LS, Andersen PS 2007 Kinetic, affinity, and diversity limits of human polyclonal antibody responses against tetanus toxoid. J. Immunol. 179, 3841–3850. (10.4049/jimmunol.179.6.3841) 17785821

[70] Li H, Ye C, Ji G, Han J 2012 Determinants of public T cell responses. Cell *Res.* 22, 33–42. (10.1038/cr.2012.1) PMC3351923 22212481

[71] Yewdell JW, Webster RG, Gerhard WU 1979 Antigenic variation in three distinct determinants of an influenza type A haemagglutinin molecule. Nature 279, 246–248. (10.1038/279246a0) 86955

[72] Price DA et al. 2004 T cell receptor recognition motifs govern immune escape patterns in acute SIV infection. Immunity 21, 793–803. (10.1016/j.immuni.2004.10.010) 15589168

[73] Schotte L, Thys B, Strauss M, Filman DJ, Rombaut B, Hogle JM 2015 Characterization of poliovirus neutralization escape mutants of single-domain antibody fragments (VHHs). Antimicrob. Agents Chemother. 59, 4695–4706. (10.1128/AAC.00878-15) 26014941 PMC4505203

[74] Radford AD, Dawson S, Coyne KP, Porter CJ, Gaskell RM 2006 The challenge for the next generation of feline calicivirus vaccines. Vet. Microbiol. 117, 14–18. (10.1016/j.vetmic.2006.04.004) 16698199

[75] Barclay VC, Sim D, Chan BHK, Nell LA, Rabaa MA, Bell AS, Anders RF, Read AF 2012 The evolutionary consequences of blood-stage vaccination on the rodent malaria *Plasmodium chabaudi*. PLoS Biol. 10, e1001368 (10.1371/journal.pbio.1001368) 22870063 PMC3409122

[76] Austin DA, Robertson PAW, Austin B 2003 Recovery of a new biogroup of *Yersinia ruckeri* from diseased rainbow trout (*Oncorhynchus mykiss*, Walbaum). Syst. Appl. Microbiol. 26, 127–131. (10.1078/072320203322337416) 12747420

[77] O'Brien KL et al. 2007 Effect of pneumococcal conjugate vaccine on nasopharyngeal colonization among immunized and unimmunized children in a community-randomized trial. J. Infect. Dis. 196, 1211–1220. (10.1086/521833) 17955440

[78] Lipsitch M 1997 Vaccination against colonizing bacteria with multiple serotypes. Proc. Natl Acad. Sci. USA 94, 6571–6576. (10.1073/pnas.94.12.6571) 9177259 PMC21091

[79] Weinberger DM, Malley R, Lipsitch M 2011 Serotype replacement in disease after pneumococcal vaccination. Lancet 378, 1962–1973. (10.1016/S0140-6736(10)62225-8) 21492929 PMC3256741

[80] Zanetti AR, Tanzi E, Manzillo G, Maio G, Sbreglia C, Caporaso N, Thomas H, Zuckerman AJ 1988 Hepatitis B variant in Europe. Lancet 2, 1132–1133. (10.1016/S0140-6736(88)90541-7) 2460710

[81] Sheldon J, Soriano V 2008 Hepatitis B virus escape mutants induced by antiviral therapy. J. Antimicrob. Chemother. 61, 766–768. (10.1093/jac/dkn014) 18218641

[82] Kallonen T, He Q 2009 *Bordetella pertussis* strain variation and evolution postvaccination. Expert Rev. Vaccines 8, 863–875. (10.1586/erv.09.46) 19538113

[83] Warfel JM, Zimmerman LI, Merkel TJ 2014 Acellular pertussis vaccines protect against disease but fail to prevent infection and transmission in a nonhuman primate model. Proc. Natl Acad. Sci. USA 111, 787–792. (10.1073/pnas.1314688110) 24277828 PMC3896208

[84] Bouchez V, Brun D, Cantinelli T, Dore G, Njamkepo E, Guiso N 2009 First report and detailed characterization of *B. pertussis* isolates not expressing pertussis toxin or pertactin. Vaccine 27, 6034–6041. (10.1016/j.vaccine.2009.07.074) 19666155

[85] Hegerle N, Guiso N 2014 *Bordetella pertussis* and pertactin-deficient clinical isolates: lessons for pertussis vaccines. Expert Rev. Vaccines 13, 1135–1146. (10.1586/14760584.2014.932254) 24953157

[86] Martin SW et al. 2015 Pertactin-negative *Bordetella pertussis* strains: evidence for a possible selective advantage. Clin. *Infect. Dis.* 60, 223–227. (10.1093/cid/ciu788) 25301209

[87] Safarchi A, Octavia S, Luu LDW, Tay CY, Sintchenko V, Wood N, Marshall H, McIntyre P, Lan R 2015 Pertactin negative *Bordetella pertussis* demonstrates higher fitness under vaccine selection pressure in a mixed infection model. Vaccine 33, 6277–6281. (10.1016/j.vaccine.2015.09.064) 26432908

[88] Woods RJ, Read AF 2015 Clinical management of resistance evolution in a bacterial infection: a case study. Evol. Med. Public Health 2015, 281–288. (10.1093/emph/eov025) 26454762 PMC4629395

[89] Andersson DI, Hughes D 2014 Microbiological effects of sublethal levels of antibiotics. Nat. Rev. Microbiol. 12, 465–478. (10.1038/nrmicro3270) 24861036

[90] D'Costa VM et al. 2011 Antibiotic resistance is ancient. Nature 477, 457–461. (10.1038/nature10388) 21881561

[91] Lavine JS, King AA, Bjørnstad ON 2011 Natural immune boosting in pertussis dynamics and the potential for long-term vaccine failure. Proc. Natl Acad. Sci. USA 108, 7259–7264. (10.1073/pnas.1014394108) 21422281 PMC3084147

[92] Gupta S, Ferguson N, Anderson R 1998 Chaos, persistence, and evolution of strain structure in antigenically diverse infectious agents. Science 280, 912–915. (10.1126/science.280.5365.912) 9572737

[93] Cobey S, Lipsitch M 2012 Niche and neutral effects of acquired immunity permit coexistence of pneumococcal serotypes. Science 335, 1376–1380. (10.1126/science.1215947) 22383809 PMC3341938

[94] Koff WC, Burton DR, Johnson PR, Walker BD, King CR, Nabel GJ, Ahmed R, Bhan MK, Plotkin SA 2013 Accelerating next-generation vaccine development for global disease prevention. Science 340, 1232910 (10.1126/science.1232910) 23723240 PMC4026248

[95] Carrat F, Flahault A 2007 Influenza vaccine: the challenge of antigenic drift. Vaccine 25, 6852–6862. (10.1016/j.vaccine.2007.07.027) 17719149

[96] Corti D, Lanzavecchia A 2013 Broadly neutralizing antiviral antibodies. Ann. *Rev. Immunol.* 31, 705–742. (10.1146/annurev-immunol-032712-095916) 23330954

[97] Euler Z, Bunnik EM, Burger JA, Boeser-Nunnink BD, Grijsen ML, Prins JM, Schuitemaker H 2011 Activity of broadly neutralizing antibodies, including PG9, PG16, and VRC01, against recently transmitted subtype B HIV-1 variants from early and late in the epidemic. J. Virol. 85, 7236–7245. (10.1128/JVI.00196-11) 21561918 PMC3126573

