# Supplementary material for: Why does drug resistance readily evolve but vaccine resistance does not?
Source: Proc Biol Sci. 2017 Mar 29;284(1851):20162562. doi: 10.1098/rspb.2016.2562 (PMC5378080; doi:10.1098/rspb.2016.2562)
Supplement: Appendix from Why does drug resistance readily evolve but vaccine resistance does not? [file rspb20162562supp1.pdf]

Electronic supplementary material: Why does drug resistance readily evolve but vaccine resistance does not?

David A. Kennedy, Andrew F. Read

1. **Mathematical framework**
2. **Schematic of treatment mosaics**

1. Mathematical framework

Here we construct mathematical models that describe resistance emergence against various interventions. We use these models to derive distributions of times until resistance emerges for interventions that have or lack early action and multiple target sites. We find that the combined effect of early action and a high multiplicity of target sites greatly reduces the rate at which resistance emerges, relative to interventions that lack one or both of these features.

To begin, we posit four scenarios (summarized in **Table S1**): a prophylactic intervention with a single effector (A), a prophylactic intervention with multiple effectors (B), a therapeutic intervention with a single effector (C), and a therapeutic intervention with multiple effectors (D). We ask how these different interventions are expected to differ in the rates at which resistance first appears.

To simplify the problem, we make six assumptions. First, by removing the transmission advantage of partial resistance (see **Figure 2**), incomplete resistance does not transmit when using prophylactic interventions (A and B), but it does transmit when using therapeutic interventions (C and D).

Second, epistatic interactions do not occur between resistance mutations. Below we discuss how this assumption could be relaxed. Third, evolution through the neutral force of genetic drift can be ignored. Fourth, selection is extremely strong such that any new resistance capable of transmission instantly becomes fixed in the population. Fifth, the probability of mutation towards resistance is both independent of the current level of resistance and equal for every replicative event, where a replicative event is defined as one pathogen cell or virion becoming two. Sixth, the probability of multiple resistance mutations occurring within a single replicative event is sufficiently small that it can be ignored when other pathways to resistance exist. Note that the qualitative ranking we give below would be unchanged if any of these last four assumptions were violated.

Given these assumptions, we can construct a model that describes the number of replicative events until resistance appears. Consider each replicative event to be a toss of a weighted coin where the two possible outcomes are 1) a mutation occurs that confers some degree of resistance, or 2) no resistance is gained. If we knew that r mutational events were required to

achieve complete resistance, and that the probability of one of these events occurring in any particular replicative event was p, then the total number or replicative events at which complete resistance first appears would be r plus a negative binomially distributed random variable with parameters r and p. It then follows from the mean of a negative binomial distribution that the average number of replicative events until this evolutionary process results in complete resistance to an intervention would be $\mu = \frac{r}{p}$. Note that in some cases, there may be two potential pathways to resistance. Complete resistance might emerge during a single replicative event, or it might emerge after an accumulation of a series of mutations that each confer partial resistance. We use this model formulation to compare the four posited interventions above.

For Intervention A, where transmission of incomplete resistance does not occur, resistance will only emerge through one of the two pathways: a single mutation that confers complete resistance. The probability of resistance occurring in any single replicative event could then be described by $p_A = \epsilon\beta$, where β is the probability that a single replicative event will result in a mu-

tation that confers some level of resistance to a particular effector, and ϵ is the fraction of those mutations that confer complete resistance to that effector. Because Intervention A uses only a single effector, only a single mutation is required for complete resistance ($r_A = 1$). The number of replicative events until resistance appears would then be the random variable W, where $W - 1 \sim \text{NB}(1, \epsilon\beta)$, and thus the average number or replicative events until the emergence of resistance to Intervention A would be $\mu_A = \frac{1}{\epsilon\beta}$.

For Intervention B, resistance must again emerge during a single replicative event to spread through the pathogen population. However, in contrast to Intervention A, N resistance mutations must simultaneously occur for resistance to be achieved. The probability of resistance being acquired in a single replicative event would therefore be reduced to $p_B = (\epsilon\beta)^N$, while the number of mutational events will remain the same, $r_B = 1$. Thus the number of replicative events until resistance appears would be the random variable X, where $X - 1 \sim \text{NB}(1, (\epsilon\beta)^N)$, giving an average of $\mu_B = \frac{1}{(\epsilon\beta)^N}$. Note that for any value of N greater than 1 and $\epsilon\beta$ less than 1, resistance to Intervention A will appear after fewer events on average than resistance to Intervention

B.

For Intervention C, where partial resistance can be transmitted, complete resistance can be acquired through an accumulation of mutations that each confers partial resistance. Using the same parameter definitions as above, the probability of a partially resistant mutation occurring during a single replicative event would be $p_C = (1 - \epsilon)\beta$, and multiple mutations would be needed to confer complete resistance, $r_C = \gamma$. The number of replicative events until complete resistance is acquired through an accumulation of partial resistance mutation would thus be the random variable U, where $U - \gamma \sim \text{NB}(\gamma, (1-\epsilon)\beta)$. A key point is that resistance could also be acquired through a single mutation of large effect, identically to that described for Intervention A. Thus the number of replicative events until resistance emerges to Intervention C would be $Y = \min(W, U)$. The dependence of W and U on the parameters ϵ and γ implies that when $\epsilon << \frac{1}{1+\gamma}$, resistance will tend to emerge through the accumulation of mutations each conferring partial resistance, resulting in $\mu_C << \mu_A$. In contrast, when $\epsilon >> \frac{1}{1+\gamma}$, resistance will tend to emerge through a single mutation that confers complete resistance,

resulting in $\mu_C \approx \mu_A$ (**Figure S1**). Although we are unable to write a closed form solution for μ_C, we are able to give it an upper bound. First, note that μ_C must of course be at least as small as μ_A. Second, note that regardless of whether a mutation confers partial resistance or complete resistance, Intervention C will have failed by the time γ of them have occurred. We can therefore conclude that $\mu_C \leq \min(\frac{\gamma}{\beta}, \frac{1}{\epsilon\beta})$.

For Intervention D, where the transmission of partial resistance can occur, and where there are N independent effectors, resistance to any single target would appear after Y replicative events identically to that for Intervention C. However, resistance would need to appear against each of N effectors before full resistance was achieved. The number of replicative events until resistance emerged against all of the effectors, would thus be the maximum of N realizations of Y. It thus follows that on average Intervention C will fail after fewer replicative events than Intervention D. Again, we are unable to write a closed form solution for the average number of replicative events until complete resistance is achieved μ_D, but we can generate an upper bound. Noting that the maximal element of a set of non-negative numbers will be

less than or equal to the sum of those numbers, and that the sum of multiple negative binomial random variables with the parameters r_i and p will itself have a negative binomial random distribution with the parameters $\sum_i r_i$ and p, the mean of the maximum of N realizations of Y must be less than or equal to N times the mean of Y. An upper bound of μ_D is therefore $N\mu_C$. This can be rewritten, $\mu_D \leq \min(\frac{N\gamma}{\beta}, \frac{N}{\epsilon\beta})$.

We can quantify the evolutionary benefit of a prophylactic intervention with multiple effectors by dividing the mean number of replicative events until the failure of each intervention, by that of Intervention B, μ_B. This analysis demonstrates that complete resistance against Intervention B would take on the order of $\frac{1}{(\epsilon\beta)^{N-1}}$ times as many replicative events to fail than any of the other three interventions (**Table S1**). Given that β is presumably much less than one, an intervention that employs both prophylaxis and multiple effectors will drastically slow the evolution of resistance relative to an intervention that lacks either or both of these features (**Figure S1**). We can thus conclude that prophylaxis and multiple effectors have a synergistic benefit in slowing the evolution of resistance to an intervention.

Note that the ordering given above, $\mu_C \leq \mu_A \leq \mu_B$ and $\mu_C \leq \mu_D \leq \mu_B$, is based on the number of replicative events until resistance appears. However, a second consideration is that the number of replicative events within a given host will be much smaller for interventions that keep pathogen population sizes small (Interventions A and B) than those that allow pathogen population sizes to become large (Interventions C and D). This could have a tremendous effect on the rate at which resistance evolves, because the mean replicative events measured above must be divided by the number of replications per infected host to determine the average number of hosts that can be infected before an intervention fails. Pathogen population sizes can vary by orders of magnitude between prophylactic and therapeutic interventions, and this could in turn play out as a potentially enormous difference in the speed of evolution.

A final point is that a resistant strain will only persist if it is able to find hosts to infect. If different hosts respond to the same intervention in different ways and a single effector provides protection, this mosaic of host responses may prevent the spread of infection (**Figure S2**). Consider an intervention

with N effectors that each confer protection in one of k ways. If hosts have equal probability of responding in any of these k ways, then using the binomial distribution, the probability that a pathogen fully resistant to intervention in one host will also be fully resistant to intervention in the next host is $(1/k)^N$. If a fraction f of the population is receiving the intervention, this strain will only persist when $R_0(1-f+f/(k^N)) > 1$, where R_0 is the effective reproductive number of the pathogen in a fully susceptible population. Thus a pathogen resistant to intervention in one host would be unlikely to persist in a host population if the coverage of the intervention f, the number of effectors N, and the number of ways that hosts can respond k were large.

In constructing our model, we made six simplifying assumption. It is possible, however, to construct similar models that relax most of these assumptions. If selection for partial resistance were present but weaker against prophylactic interventions than therapeutic interventions, our qualitative conclusion that $\mu_C \leq \mu_A \leq \mu_B$ and $\mu_C \leq \mu_D \leq \mu_B$ would still hold, but the absolute benefit of prophylaxis would be reduced. In practice, this model could be achieved by adding a pathway to complete resistance for prophy-

lactic interventions through the accumulation of partial resistance (similar to that for the therapeutic interventions). In this new model, an additional parameter ψ would describe the ability of selection to act on partial resistance for prophylactic interventions relative to therapeutic interventions. The assumption of no epistatic interactions could be relaxed by reformulating the model, where the precise form of the new model would depend on the nature of the epistatic interactions. For example, an ordered stepwise evolution of resistance model could be modeled as the sum of several geometrically distributed random variables, each with a potentially different value for the parameter p that depends on the mutation rate for each step of this evolutionary process. For other types of epistatic interactions, other model formulations might be needed. Depending on the specific details of the epistatic interactions, the failure rates of Interventions C and D could be dramatically increased or reduced. Genetic drift could be added to the model by including an extra pathway to resistance through neutral drift, although the probability of following this pathway to resistance is likely to be very small. The assumption that selection is extremely strong could be relaxed by explicitly

modeling the spread of resistance mutants through the pathogen population, or this process could be approximated by adding the expected fixation times to the number of replicative events. It is difficult to imagine an easy way to relax our final two assumptions that the probability of a mutation towards resistance is constant for any replicative event and that the probability of two resistance mutations occurring during the same replicative event is very small. These two assumptions, however, have no effect on the qualitative ordering that $\mu_C \leq \mu_A \leq \mu_B$ and $\mu_C \leq \mu_D \leq \mu_B$. They also seem likely to have little influence on the quantitative relationship between the interventions when considering biologically reasonable parameter space.

Table S1: The effects of Interventions A-D on the evolution of resistance.

**Intervention**	**Multiplicity of effectors**	**Prophylaxis**	**Replicative events until resistance**	**Mean replicative events until resistance**	**Mean replicative events relative to Intervention B**
Intervention A	One	Yes	W, where:<br>$W-1 \sim \mathrm{NB}(1, \epsilon\beta)$	$\mu_A = \frac{1}{\epsilon\beta}$	$\frac{\mu_A}{\mu_B} = (\epsilon\beta)^{N-1}$
Intervention B	Many	Yes	X, where:<br>$X-1 \sim \mathrm{NB}(1, (\epsilon\beta)^N)$	$\mu_B = \frac{1}{(\epsilon\beta)^N}$	$\frac{\mu_B}{\mu_B} = 1$
Intervention C	One	No	Y, where:<br>$Y = \min(W, U)$,<br>$U - \gamma \sim \mathrm{NB}(\gamma, (1-\epsilon)\beta)$	$\mu_C \leq \min(\frac{\gamma}{\beta}, \frac{1}{\epsilon\beta})$	$\frac{\mu_C}{\mu_B} \leq \min(\gamma\epsilon^N\beta^{N-1}, (\epsilon\beta)^{N-1})$
Intervention D	Many	No	Z, where:<br>Z=max$(Y_1, Y_2, \ldots, Y_N)$	$\mu_D \leq \min(\frac{N\gamma}{\beta}, \frac{N}{\epsilon\beta})$	$\frac{\mu_D}{\mu_B} \leq \min(N\gamma\epsilon^N\beta^{N-1}, N(\epsilon\beta)^{N-1})$

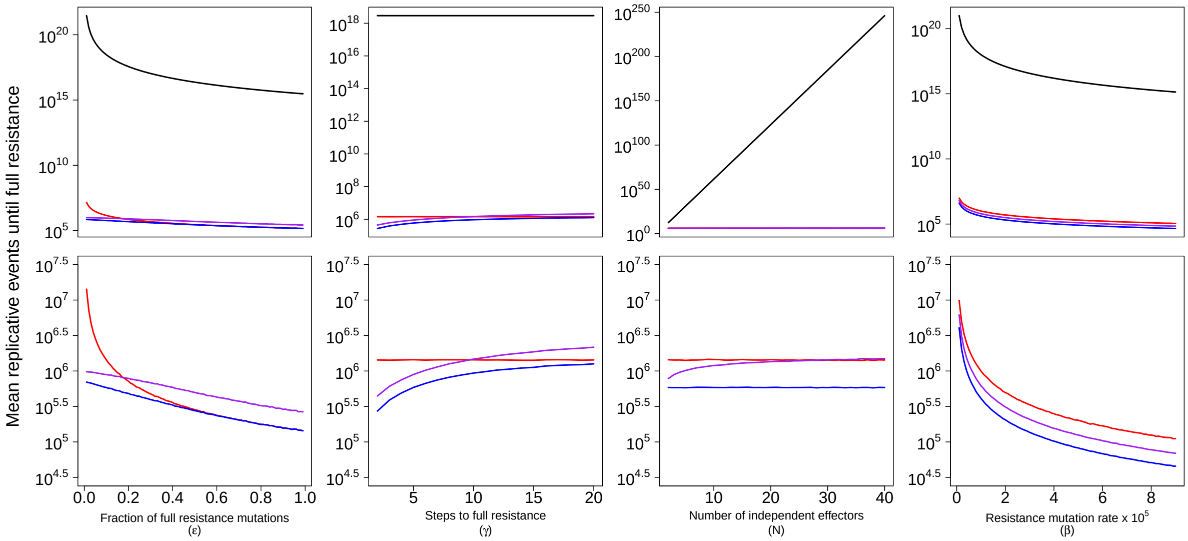

Figure S1: Mean replicative events until resistance appears for Interventions A (red), B (black), C (blue), and D (purple). Plotted data is the mean of 20000 realizations for each parameter set and model. Each column shows the effect of varying a different model parameter. The bottom row shows the same data as the top row, magnified to highlight the differences between Interventions A, C and D. Note that for all parameter sets the average number of replicative events until resistance appears shows $\mu_C \leq \mu_A \leq \mu_B$ and $\mu_C \leq \mu_D \leq \mu_B$. The relationship between A and D depends on the parameters ϵ, γ, and N. Where unspecified, $\beta = 7 \times 10^{-6}$, $\epsilon = 0.1$, $N = 3$, $\gamma = 5$.

2. Schematic of treatment mosaics

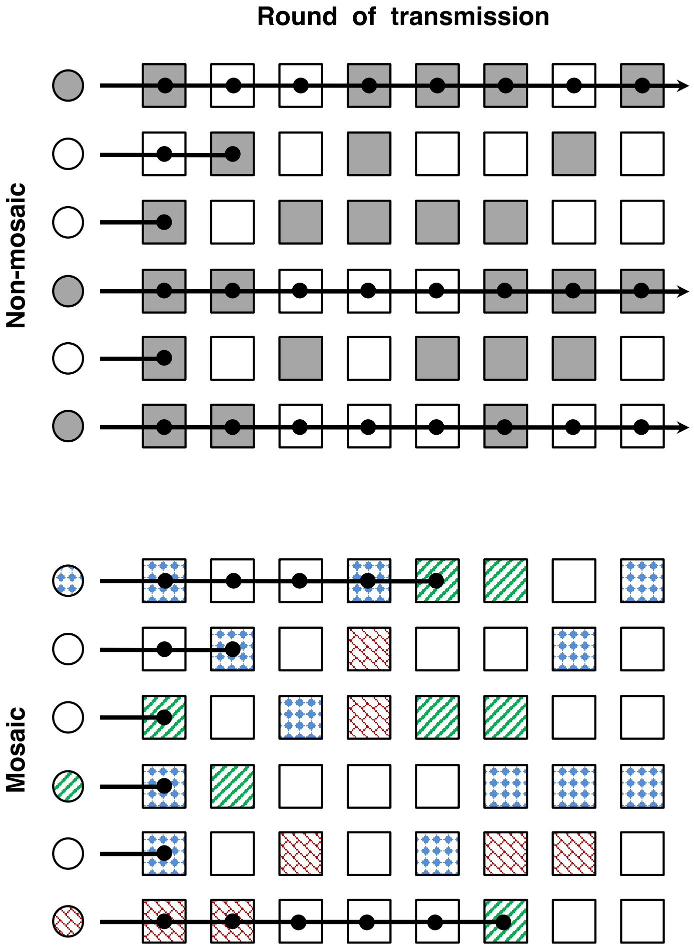

Figure S2: Schematic showing the effect of treatment mosaics on pathogen resistance. White boxes are untreated hosts. Filled and patterned boxes are treated hosts, with different colors and patterns denoting different mechanisms of actions. Circles denote pathogens, with the different colors and patterns indicating resistance to a particular mechanism of action. Open circles are fully susceptible pathogens. Each line shows a chain of transmission that ends when the pathogen infects a host with an effective mechanism of action. Note that the overall levels of resistance and intervention are the same between the non-mosaic situation (top) and the mosaic situation (bottom). The success of a fully susceptible pathogen is therefore the same in both situations, but the resistant pathogens are less successful in the presence of a mosaic. As a result, mosaics reduce the strength of selection for resistance to a particular mechanism of action, because selection acts on the relative success of the different virus strains.
